# Shifting Syllable Production in an Ex Situ Population of a Critically Endangered Songbird

**DOI:** 10.1002/zoo.70027

**Published:** 2025-10-06

**Authors:** Oliver Jepson, R. T. Gilman, Leah J. Williams, Rebecca N. Lewis

**Affiliations:** ^1^ Department of Earth and Environmental Sciences, Faculty of Science and Engineering University of Manchester Manchester UK; ^2^ Chester Zoo, Upton‐by‐Chester Chester UK

**Keywords:** anthropogenic disturbance, Bali myna, behavioural plasticity, birdsong

## Abstract

Singing is an ecologically important behaviour for songbirds. Syllables function as the building blocks of birdsong, so changes to their production will have implications for overall song structure. It is well established that anthropogenic disturbance can influence syllable production in wild songbird populations, but the effect of anthropogenic disturbance on syllable production in ex situ populations has not been studied. We set out to fill this gap by comparing the syllable production of Chester Zoo's Bali myna (*Leucopsar rothschildi*) population during a period of zoo closure in 2020 (due to the COVID‐19 lockdown) to a period of normal zoo opening in 2019. The number of syllables per song, the rate at which syllables were produced and the diversity of syllables all showed evidence of plasticity across days and years. However, only the number of syllables per song responded significantly to anthropogenic disturbance. Changes in syllable number due to anthropogenic disturbance could mitigate potential signal masking from unpredictable noise, although communication efficacy may still be affected. As a result, changes in vocal communication could impact conservation breeding programmes by altering the way that individuals interact with conspecifics.

## Graphical abstract

Bali mynas produced songs containing more syllables under increased anthropogenic disturbance.

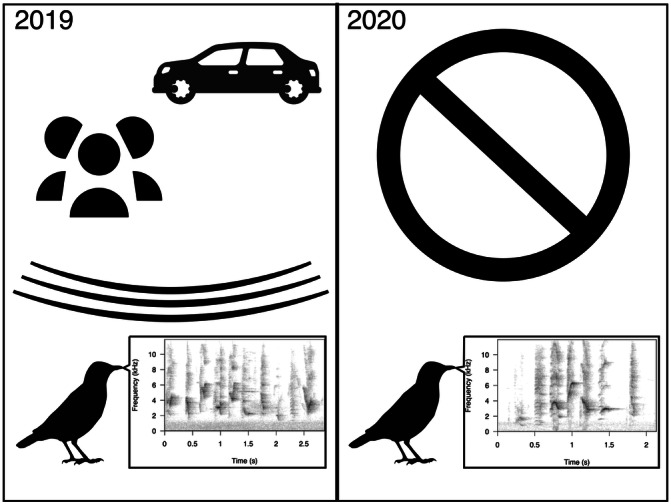

## 1 Introduction

Birdsong has well‐documented roles in mate selection, territory defence and maintenance, and the communication of aggression (Marler and Slabbekoorn 2004; Collins 2004; Illes et al. 2006; DuBois et al. 2009). Therefore, it is important to understand the factors that drive changes in birdsong, as these could impact the behavioural ecology of species in conservation settings. Multiple factors can affect birdsong, including social learning, morphology and aspects of the environment such as the soundscape. In human**‐**dominated environments, anthropogenic disturbance is known to impact birdsong (e.g., Slabbekoorn and Peet 2003; Potvin et al. 2011; Winandy et al. 2021). Although not often considered, understanding the effects of anthropogenic disturbance on birds' vocal behaviour in zoo environments is important, as many of the bird species in zoos are endangered and are valuable parts of ex situ conservation efforts (Lewis et al. 2021).

Birdsong is composed of syllables—continuous sounds separated by short silences (Harma 2003). Birds can respond to the order, rate and spectral characteristics of the syllables that they hear (Comins and Gentner 2014; Hoi et al. 2023; Fishbein et al. 2020; Leitner et al. 2001). Therefore, environmental factors that change how birds produce syllables, or which syllables they produce, may affect how birds communicate and interact. A key component of anthropogenic disturbance is anthropogenic noise, which is typically continuous, low‐frequency and often traffic‐derived. Such noise can drive birds to sing songs containing fewer syllables (e.g., Verzijden et al. 2010; Potvin et al. 2011; Cartwright et al. 2014) with lower syllable diversities (e.g., Montague et al. 2013; Winandy et al. 2021), delivered at different rates (syllables per second) (e.g., Potvin et al. 2011). Furthermore, the syllables themselves are sometimes longer in noisier conditions (e.g., Bermúdez‐Cuamatzin et al. 2011). Some of the syllable attributes that change in the presence of anthropogenic noise have been linked to ecological roles such as aggression communication during territory defence (DuBois et al. 2009).

In zoos, anthropogenic noise is not limited to continuous, low‐frequency mechanical noise. Human speech, from visitors and staff, covers a broad range of frequencies (Fant 2007) and may be more unpredictable than mechanical noise. Temporary sounds from enclosure maintenance may also contribute to the soundscape (Lewis et al. 2024). As a result, the acoustic conditions ex situ populations are exposed to differ compared to most in situ populations. In addition to zoo visitors' contribution to the soundscape, the physical presence of visitors has been known to affect behaviour in many taxa (Sherwen and Hemsworth 2019) and could impact syllable production in birds.

Whilst vocal change in ex situ populations has been reported in several species (Hawaiian crows (*Corvus hawaiiensis*) (Tanimoto et al. 2017), regent honeyeaters (*Anthochaera phrygia* (*A. phrygia*)) (Crates et al. 2021) and golden mantella frogs (*Mantella aurantiaca*) (Passos et al. 2017, 2021)), the cause of these changes remains poorly understood. Other work has linked auditory enrichment to changing vocalization rates in several bird species (Robbins and Margulis 2016; Williams et al. 2017). However, these studies did not investigate song structuring. Furthermore, while the enrichment itself featured sounds that could equate to anthropogenic disturbance, such as music and a talking radio, these studies did not consider other potential sources of disturbance. Therefore, we know little about how anthropogenic disturbance contributes to vocal communication in ex situ populations.

Bali mynas (*Leucopsar rothschildi*) are a critically endangered species that currently depends on ex situ conservation (BirdLife International 2023). They are monogamous and form long‐term pair bonds. Outside of their breeding season, they live socially and aggregate into flocks. This behaviour is lost during the breeding season, when pairs assume territories (Craig and Feare 2010). Both sexes use visual and vocal displays to communicate defence of their territory to intruders and to court each other. During the visual aspect of these displays, the birds raise their crests, point their beaks upwards and bob their bodies. This is accompanied by singing (San Diego Zoo Wildlife Alliance 2024), making vocal behaviour an important part of both sexes' behavioural ecology. Thus, changes in vocal behaviour, including syllable production, during ex situ breeding could have implications for the species' management and conservation (Lewis et al. 2021; Appleby et al. 2023).

In 2020, a closure of Chester Zoo due to the COVID‐19 pandemic resulted in the absence of visitors and an altered soundscape compared to periods when the zoo was open (Lewis et al. 2024). This created an opportunity to study birdsong in the zoo's ex situ populations under conditions of low and high anthropogenic disturbance, where ‘anthropogenic disturbance’ includes both anthropogenic noise and the presence of visitors in the zoo.

To investigate the effect of anthropogenic disturbance on syllable production in the Bali myna population at Chester Zoo, we compared syllable number (i.e., syllables per song), syllable rate (i.e., syllables per second in each song) and the diversity of syllables in two time blocks (‘morning’ and ‘afternoon’) in 2019 and 2020. The zoo was closed during the ‘morning’ time block in both years. In the ‘afternoon’ time block, the zoo was open in 2019 but closed in 2020, resulting in a larger increase in disturbance between the ‘morning’ and ‘afternoon’ periods in 2019. We expected that syllable production would change over the course of the day, regardless of anthropogenic influence. However, if anthropogenic disturbance affects syllable production in this species, then we would expect the way in which syllable production changes through the day to differ between the zoo opening period in 2019 and the closure period in 2020.

## 2 Materials and Methods

### 2.1 Study Site and Population

This study uses recordings taken from the Bali Temple aviary at Chester Zoo, UK. This is an outdoor, walkthrough aviary situated at the periphery of the zoo, near a main road (the A41). A visitor path travels through the aviary, with visitors and birds occupying the same space without a barrier. Sound produced by visitors (voices, footsteps, etc.) and traffic noise from the nearby road both contribute to the soundscape in the aviary.

The Bali myna population that we studied was all‐female and consisted of nine birds in 2019, decreasing to five birds in 2020. All the birds present in 2020 were present in 2019. The population was housed with a mix of Southeast Asian bird species, which underwent a change in species composition between the years (see Lewis et al. 2024 for species lists). The total number of birds in the aviary increased from 73 in 2019 to 83 in 2020.

### 2.2 The Recordings

Recordings were taken from two time periods (4th–7th May 2019 and 9th–12th April 2020) using a Wildlife Acoustics SM4 recorder with inbuilt omnidirectional microphones (Wildlife Acoustics Inc., Maynard, MA, USA), a 24‐kHz sampling rate and a 16‐bit recording depth. The recording equipment was placed in a planted area near to the visitor path, and the position of the recording equipment within the aviary was consistent across the years. The recordings used for this study are a subset of those studied by Lewis et al. (2024), where more detailed information on sampling procedures is available.

During the recording period in 2019, the zoo was open to the public in the afternoon, and the levels of traffic on the nearby road were normal. In 2020, the UK's COVID‐19 lockdown resulted in temporary zoo closure and reduced the movement of people, causing a 70% reduction in road usage (GOV.UK 2023). The zoo closure period significantly impacted the soundscape in the Bali Temple aviary. Overall sound pressure levels (dB(Z)) were lower in 2020 compared to 2019, with a particular reduction in low‐frequency (17.6–890.9 Hz) noise (Lewis et al. 2024). Additionally, visitors were completely absent due to the zoo's closure. These differences enabled us to compare songs and their constituent syllables in contrasting soundscapes and states of visitor presence.

### 2.3 Analysis of the Recordings

The hours of 07:47–08:47 and 13:47–14:47 BST (13:48–14:21 in one instance) were selected for analysis in 2019, and 08:48–09:48 and 13:48–14:48 BST were selected in 2020 (‘morning’ and ‘afternoon’ hereafter). The differing morning times resulted in similar proximity of samples to sunrise in each study period, with average sunrise times of 05:26 in 2019 and 06:19 in 2020. This was important for standardising the effects of the dawn chorus on results. Four days were sampled in each year. We repeated our analyses matching the morning civil times in each year (07:47–08:47 in 2019 and 07:48–08:48 in 2020), with results remaining qualitatively similar in most cases (SI 1). In the mornings, mean sound pressure levels were approximately 66 dB(Z) in 2019 and 62 dB(Z) in 2020. In 2019, sound pressure levels in the afternoon increased to a mean level of 72.5 dB(Z). In 2020, sound levels in the afternoon period were similar to those in the morning period and lower than the afternoon period in 2019, with a mean sound pressure level of ~63 dB(Z) (Lewis et al. 2024). If anthropogenic disturbance affects syllable production, we would expect daily changes in syllable production characteristics to differ between the years due to differences in disturbance levels during the afternoon periods in each year.

**Figure 1:**
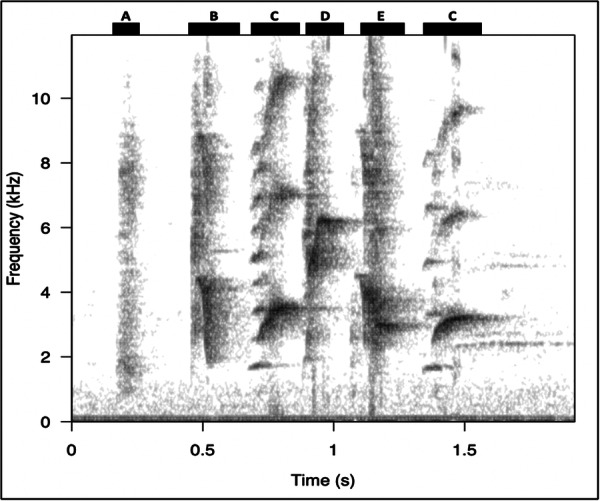
A spectrogram of a Bali myna song considered suitable for analysis. The lettering above the bars at the top of the figure shows how the syllables in this song may be classified. The spectrogram was created using Seewave (Sueur et al. 2008) (sample rate = 24 kHz, window length = 512, overlap = 90%).

Songs were extracted from the recordings and analysed using the web‐based bioacoustics analysis software Koe (Fukuzawa et al. 2020). We defined songs as series of syllables with inter‐syllable intervals (temporal gaps between syllables) of < 1 s (Kagawa and Soma 2013). The syllables in the songs had to leave clearly visible traces on a spectrogram (e.g., Figure 1) to be considered suitable for analysis. Examples of unsuitable songs include those that were heavily obscured by masking from other sounds, too quiet to distinguish syllables, or contained multiple Bali mynas singing at similar amplitudes (in which case songs were difficult to separate in the recordings). Once all suitable songs were extracted from the recordings, songs shorter than six syllables were removed (see Table 1) to avoid including contact calls in the analyses. This is important because, in many species, contact calls are structurally different (often shorter and less diverse) than songs (Marler 2004). The usage rate of contact calls might change over the course of the day (Hillemann et al. 2019), and we did not want to confound changes in call type with changes in syllable production in songs. The threshold of six syllables defining a song in our study is conservative; we expect contact calls to be shorter than six syllables, based on observation, probably around 3–4 syllables, but we used a higher threshold because calling behaviour is not well studied in this species.

**Table 1:** Sample sizes of songs and syllables from the morning and afternoon in 2019 and 2020.

Year	Time block	Songs	Syllables
2019	Morning	42	384
	Afternoon	39	503
2020	Morning	145	1313
	Afternoon	200	1856

*Note:* In 2019, the zoo was closed in the morning but open in the afternoon. In 2020, the zoo was closed in the morning and afternoon. Based on personal observation, birds sung less frequently across both time periods in 2019. This resulted in less useable songs.

Syllables within songs were defined as individual sounds, or series of similar sounds, separated by silent inter‐syllable time gaps longer than ~5 ms, a commonly used criterion in other species (Sakata et al. 2008; Kershenbaum et al. 2016). Syllables were classified based on their visual similarity in the spectrogram and assigned to bins representing their ‘syllable type’ (Figure 1). To classify different syllable types, we produced a key containing exemplars and descriptions of each syllable type (SI 2) based on 24 haphazardly sampled songs. To test the inter‐observer reliability of this classification system, a second observer used this key to classify the syllables in the sampled songs. The success of their classification was then checked, and revisions were made to the classification system where common discrepancies were noted. For example, if the second observer consistently mistook two syllable types for one another, these classes were combined into one. After several revisions, an interobserver reliability of 79.3% was achieved for the classification of syllables in the sample. The resulting key was then used to classify syllables in the rest of the songs sampled, with new syllable types absent from the original key added when necessary. For each syllable in the data set, we recorded the song from which it came, the syllable type, the date of the recording, the start and end times of the syllable in its song, and the year and time block in which the syllable was produced.

### 2.4 Data Analysis

We chose three syllable production characteristics for analysis: syllable number, syllable rate and syllable diversity. We defined syllable number as the total number of syllables in a song. We subtracted six from the syllable number for each song to create an adjusted syllable number because vocalisations with six or fewer syllables were excluded from analyses. Thus, the adjusted syllable numbers began at 0 and could be studied with negative binomial regression. We defined syllable rate as the number of syllables per second in each song. We log‐normalised the syllable rate so we could study it with linear regression. To measure syllable diversity, we computed the Shannon diversity index of the syllable types within individual songs using the vegan package in R (Oksanen et al. 2022). We studied syllable diversity within songs using linear regression with Shannon's diversity as the response variable. There are many other aspects of syllable production that might be studied, but we chose these three because they are relatively insensitive to signal‐to‐noise ratio and the distance of birds from the microphone.

To determine the effects of the time block, year of recording and the interaction between them on the syllable production characteristics we studied, we used a series of regression models in R (R Core Team 2023). This interaction term tested whether each aspect of syllable production studied changed differently through the day in each year. This would suggest an effect of anthropogenic disturbance on syllable production. If significant interactions were not identified in these models, we fitted separate models studying the effect of time on data from within each year to better understand how time affected syllable production within the different years.

## 3 Results

We studied a total of 4056 syllables from 426 songs (Table 1). Across all songs, we identified 23 syllable types (Supporting Information). After excluding vocalisations with six or fewer syllables, the mean syllable number was 9.5 syllables, and songs included an average of 6.4 distinct syllable types.

In many bird species, singing behaviour changes over the course of the day (e.g., Moran et al. 2019; Dinh et al. 2020; Pérez‐Granados and Schuchmann 2020). However, if Bali myna songs are affected by anthropogenic disturbance, we would expect singing behaviour to change differently with time of day in 2020 (zoo closed) compared to 2019 (zoo open), as soundscape differences were more pronounced in 2019 than in 2020 and visitors were absent in the latter year. This hypothesis was supported for one of the three measures we studied.

**Figure 2:**
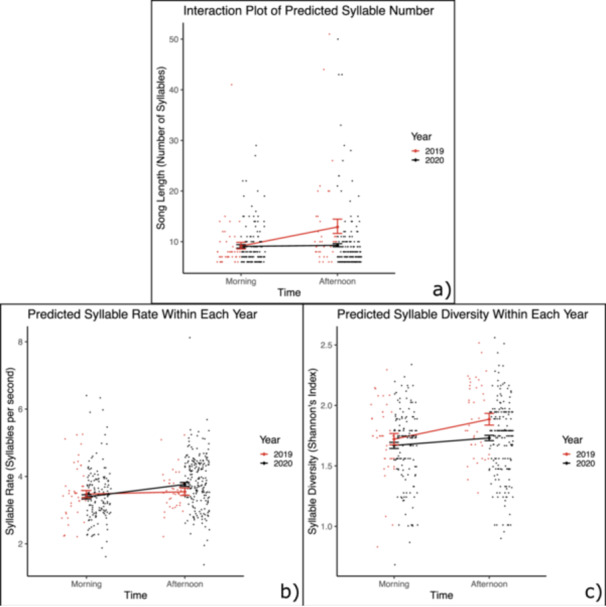
Syllable production changed differently over the course of the day in 2019 versus 2020 for one of the three variables studied. Red represents 2019, and black represents 2020. The points denoted by the larger diamonds show model predictions, and the error bars represent the standard error of these predictions. The smaller points show the raw data. The predictions from (a) are from a single model containing an interaction between year and time, while the predictions from (b) and (c) are from separate models computed for each year.

The number of syllables per song increased by 1.66 from morning to afternoon in 2019 (*p* = 0.006) but did not change between morning and afternoon in 2020 (*p* = 0.625) (*p*(interaction) = 0.0269) (Figure 2a).

We found no evidence for an overall change in syllable rate between years (*p* = 0.501) (Figure 2b). Across both years combined, birds sang 0.31 syllables s^−1^ faster in the afternoon than in the morning (*p* < 0.001). When we fit separate models to the data from each year, we found that the syllable rate increased by 0.37 syllables s^−1^ from morning to afternoon in 2020 (*p* < 0.001), but we found no evidence that the syllable rate changed from morning to afternoon in 2019 (*p* = 0.651). However, the interaction between year and time of day was not significant (*p* = 0.115).

Across the years, Shannon's diversity of the syllables within the songs increased by 0.075 from morning to afternoon (*p* = 0.013). In addition, songs from 2019 had higher Shannon's diversity than songs from 2020 by, on average, 0.10 (*p* = 0.013). We did not find evidence that syllable diversity changed significantly differently between the 2 years (*p*(interaction) = 0.175). When we fit separate models to the data from each year, we found that syllable diversity increased from morning to afternoon by 0.16 in 2019 (*p* = 0.017) and by 0.06 in 2020 (*p* = 0.067). We saw slightly different patterns in our Supporting Data, where the morning time block was 07:48–08:48. In these data, syllable diversity still increased from morning to afternoon by 0.16 in 2019 (*p* = 0.017), but there was no increase in 2020 (*p* = 0.98), and the interaction between time and year was significant (*p*(interaction) = 0.039) (see Supporting Information).

## 4 Discussion

We studied how three measures of syllable production in Bali myna songs recorded in a walk‐through aviary at Chester Zoo changed between morning and early afternoon in 2019 and 2020. In both years, the zoo was closed and relatively quiet in the mornings. In 2019, the zoo was open, and birds were exposed to visitors and other anthropogenic disturbance in the afternoons. In 2020, the zoo was closed, and birds were relatively undisturbed in the afternoons (Lewis et al. 2024). Thus, if syllable characteristics change differently between morning and afternoon in the two years, it is likely to be due to anthropogenic disturbance. Syllable number increased more from morning to afternoon in 2019 than in 2020, but the change in syllable rate over the course of the day was similar in both years. We found no evidence that daily changes in within‐song syllable diversity differed between the years in our focal data set, with both years showing increased syllable diversity in the afternoon. However, this was not the case in our Supporting Data, where an increase in syllable diversity was only observed in 2019. To our knowledge, this is the first study to investigate the behavioural responses of Bali mynas to anthropogenic disturbance, and the first to investigate the response of syllable production to anthropogenic disturbance in zoos.

Syllable number increased with anthropogenic disturbance in our study, but the opposite pattern has been noted in natural environments subjected to experimental noise near roadsides (Verzijden et al. 2010; Cartwright et al. 2014; Ríos‐Chelén et al. 2015) and in urban environments (Potvin et al. 2011). This contrast could be due to the nature of the soundscape changes associated with anthropogenic disturbance in the zoo. Noises associated with visitors, such as voices, were variable and likely resulted in more sporadic disturbance than from more consistent sources of anthropogenic noise, such as traffic. In these conditions, signal redundancy may be important. The probability of error in information transfer through a noise‐affected channel can be reduced by increasing the signal's redundancy (Shannon 1948). This approach to overcoming the challenge of communicating through noise is used by some animal species in the presence of constant noise (Brumm and Slabbekoorn 2005) and may be particularly effective under sporadic noise; if merely hearing a syllable is enough for a recipient to receive some information from a song, longer songs may be better suited to successful signal transmission as they are less prone to complete masking by brief sounds. Furthermore, human voices cover broad frequency ranges (Fant 2007), so alternative ways of avoiding masking from noise, such as syllable frequency (Hz) increases (e.g., Slabbekoorn and Peet 2003; Bermúdez‐Cuamatzin et al. 2009), may be ineffective. Further work could compare the responses of birdsong in individual species between sporadic and consistent noise to substantiate this speculation.

The interaction between time and year on syllable rate in our models was not significant, so we cannot conclude that anthropogenic disturbance affected syllable rate. However, when we studied the years separately, we found that time positively affected syllable rate in 2020 but not in 2019. This pattern is consistent with a negative effect of anthropogenic disturbance on syllable rate in 2019, which could have constrained the increase we observed from morning to afternoon in 2020. Previous work studying how anthropogenic noise in urban environments affects silvereye (*Zosterops lateralis*) song found a negative effect on syllable rate. The authors of this study suggested that singing at lower rates allows birds to better communicate in noisy environments (Potvin et al. 2011). This idea is likely also true for zoo environments and aligns with our explanation for syllable number: by reducing syllable rate, birds could increase their time spent singing and lower the chances of their songs being completely masked by sporadic sounds. Our results are consistent with this pattern, but they are not evidence, and we believe further study on this subject is warranted. Syllable rate has been linked to aggression communication in songbirds (Hoi et al. 2023), so changes in syllable rate could have implications for the outcome of intraspecific agonistic interactions and, consequently, the welfare of captive individuals.

We found different patterns for syllable diversity in the two data sets we studied. In our focal data set, we observed increased syllable diversity in the afternoons of both years and no evidence that these increases differed between years. However, when we defined the morning time block differently, we found that syllable diversity increased in the afternoon of 2019 but not 2020. The results from our two data sets are not conflicting. The pattern of greater daily change in 2019 is the same in both cases but reaches statistical significance in the Supporting Data (*p* = 0.039) and not in the focal data (*p* = 0.175). More data on Bali myna singing behaviour in disturbed and undisturbed environments will be needed to understand if or how disturbance affects within‐song syllable diversity.

Our study was opportunistic. Recording equipment was already in place when Chester Zoo closed during the pandemic. This presented a rare opportunity to compare Bali myna song in the presence of high and low levels of anthropogenic disturbance. Because the study was opportunistic, there were potentially important factors that we could not fully control. Perhaps the most important factor is that the individual Bali mynas and other bird species present in the aviary changed between 2019 and 2020. If individual Bali mynas change their syllable production in different ways over the course of the day, either due to intrinsic behaviours or due to the composition of the bird community in which they live, then these factors rather than differences in anthropogenic disturbance could explain the patterns we observed. However, it is not clear why these factors would drive differences in diel song structuring patterns, and, to the best of our knowledge, evidence that they can do so has not been documented previously. Therefore, we believe that anthropogenic disturbance, which certainly changed through the day differently in each year and has been directly linked to syllable production in the past, is the most likely driver. There were also climatic differences between the study periods that we could not control. Temperature (Coomes and Derryberry 2021) and precipitation (Lengagne and Slater 2002) have been known to affect avian vocal activity and could have influenced our results, but, again, these have not been directly linked to song structuring before. We also cannot disentangle the effects of the different aspects of anthropogenic disturbance, such as visitor presence and anthropogenic noise. However, as these sources of disturbance often occur together in zoos, our findings could still have practical use for ex situ conservation efforts. Playback of noise in the absence of visitors under similar climatic conditions could help to disentangle these factors in future studies and may be achieved by studying ex situ bird populations on days of zoo closure due to seasonal holidays and comparing their vocal activity in the days immediately following, when the zoo reopens.

Our study provides new evidence that anthropogenic disturbance may affect the number of syllables in birdsong. This may have implications for conservation. In many bird species, songs and syllables are learned, which can lead to heritable song cultures that may change in captivity (Tanimoto et al. 2017). If the changes in syllable number we observed are adaptive in the zoo environment, anthropogenic disturbance could contribute to long‐term changes in the vocal behaviour of zoo birds, resulting in divergent vocal behaviour between populations in situ and ex situ (Tanimoto et al. 2017). These differences could impact the success of conservation programmes by affecting song preference and recognition (Lewis et al. 2021). For example, zoo‐bred female regent honeyeaters (*A. phrygia*) favour the songs of zoo‐bred males over wild males (Appleby et al. 2023). As a result, zoo‐bred individuals may not be able to fully integrate into new populations if reintroduced (Lewis et al. 2021). Therefore, changes in vocal behaviour could be relevant for the conservation of the Bali myna. However, it should also be noted that the changes observed in this study appeared to be plastic. Diel patterns in vocal behaviour differed between years, suggesting that Bali myna can alter vocalizations quickly in response to environmental factors. Similar plasticity in response to soundscape change has been reported in other species. For example, when released from low‐frequency masking noise due to the COVID‐19 pandemic, white‐crowned sparrows (*Zonotrichia leucophyrs*) in urban territories began singing quieter songs with greater frequency bandwidth (Derryberry et al. 2020). If Bali mynas can alter their singing behaviour quickly in response to changing conditions, birds released from zoo populations into the wild may successfully adapt their songs to the new environmental and social conditions. This might help them overcome potential challenges associated with integration into wild populations, such as assortative mating (Lewis et al. 2021), supporting the suitability of zoo Bali myna populations as sources for reintroduction schemes.

Our results are from a small study that exploits a unique contrast provided by zoo closure during the COVID‐19 pandemic. Future work should assess potential measures to mitigate the effects of anthropogenic disturbance on ex situ songbird populations and verify whether cultural divergence between captive and wild Bali myna song has occurred. We also recommend investigating whether anthropogenic disturbance has similar effects on the song of other captive songbird species. Our study provides motivation and testable hypotheses for this study.

## Ethics Statement

This project was approved by Chester Zoo and the University of Manchester Animal Welfare and Ethics Review Board.

## Supporting information


**Supporting Material 1:** Shows the same information as in Figure 2. Red represents 2019, and black represents 2020. The points denoted by the larger diamonds show model predictions, and the error bars represent the standard error of these predictions. The smaller points show the raw data. The predictions from Figure 2b,c are from separate models computed for each year. These figures differ from Figure 2 in that the morning of 2020 is set to 07:48–08:48 as opposed to 08:48–09:48.

**Supporting Material 2:** The syllable key used to classify the syllable types in this study. The first column shows the syllable class and a brief description of its characteristics, while the second and third columns show some exemplars of that class at 100% and 400% zoom in Koe, respectively. The contrast in Koe was set to 100 for all these exemplars.

## Data Availability

The data that support the findings of this study are openly available in Figshare at https://figshare.manchester.ac.uk/, Reference Number 10.48420/27110629.
