# Supplementary figures and images for: Shifting Syllable Production in an Ex Situ Population of a Critically Endangered Songbird
Source: Zoo Biol. 2025 Oct 6;45(1):29–36. doi: 10.1002/zoo.70027 (PMC12884255; doi:10.1002/zoo.70027)

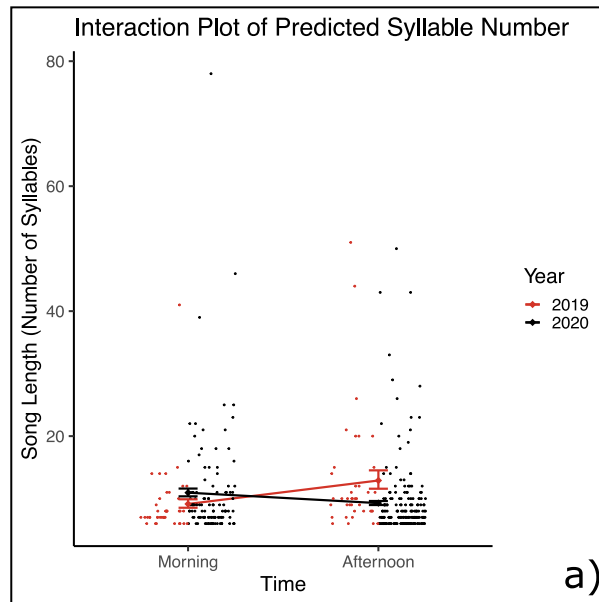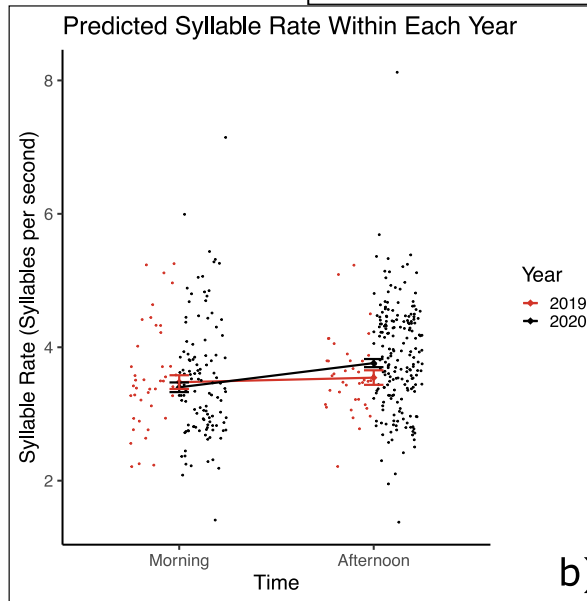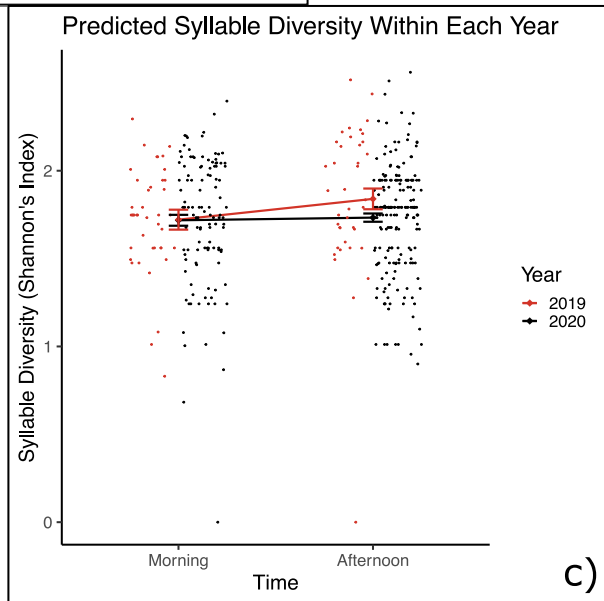

Supplement: Supplementary file 1 — Supporting Material 1: Shows the same information as in Figure 2. Red represents 2019, and black represents 2020. The points denoted by the larger diamonds show model predictions, and the error bars represent the standard error of these predictions. The smaller points show the raw data. The predictions from Figure 2b,c are from separate models computed for each year. These figures differ from Figure 2 in that the morning of 2020 is set to 07:48–08:48 as opposed to 08:48–09:48. [file ZOO-45-29-s001.pdf]
