# Supplementary material for: Shifting Syllable Production in an Ex Situ Population of a Critically Endangered Songbird
Source: Zoo Biol. 2025 Oct 6;45(1):29–36. doi: 10.1002/zoo.70027 (PMC12884255; doi:10.1002/zoo.70027)
Supplement: Supplementary file 2 — Supporting Material 2: The syllable key used to classify the syllable types in this study. The first column shows the syllable class and a brief description of its characteristics, while the second and third columns show some exemplars of that class at 100% and 400% zoom in Koe, respectively. The contrast in Koe was set to 100 for all these exemplars. [file ZOO-45-29-s002.pdf]

	100%	400%
A<br><br>Short chaotic notes	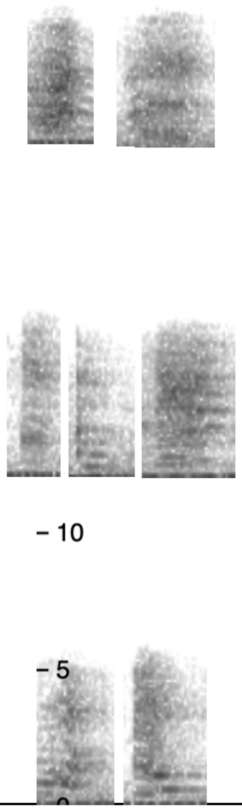	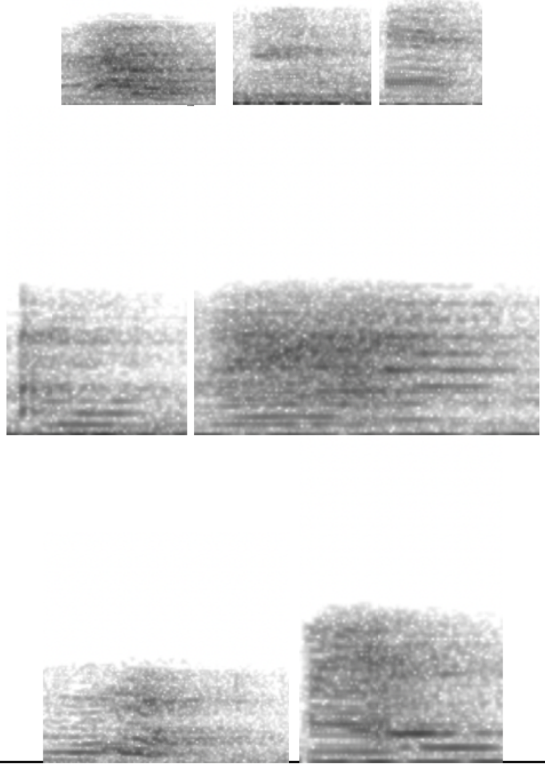

B<br>Low frequency flat (<100 ms)	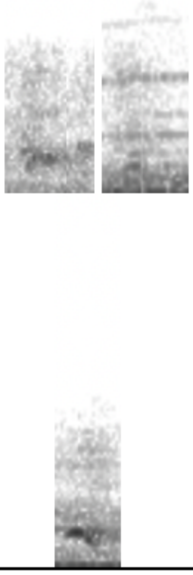	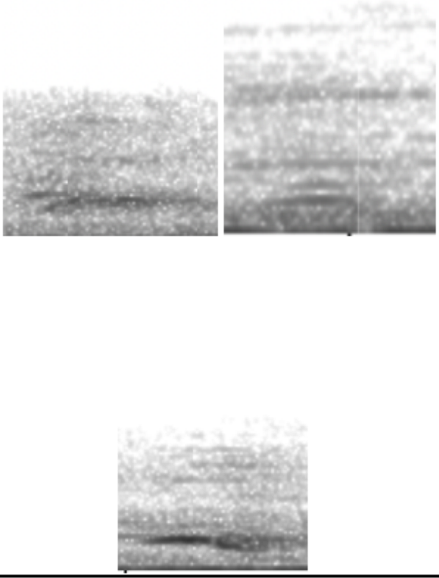
C<br><br>Low frequency flat (>100 ms)	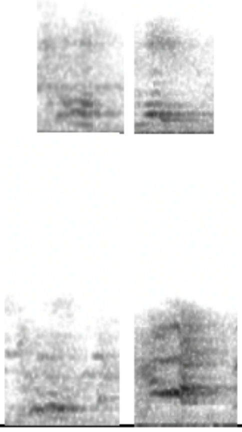	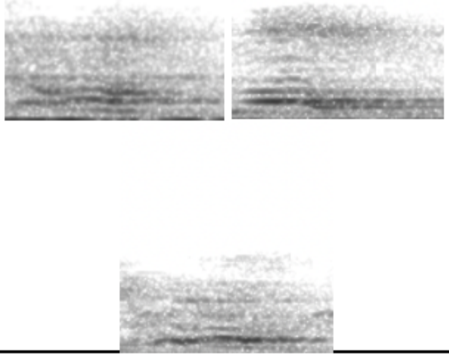

	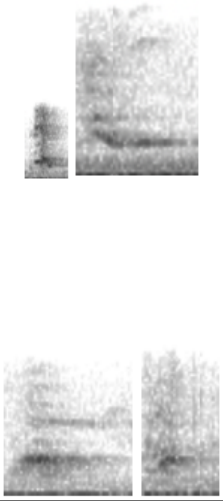	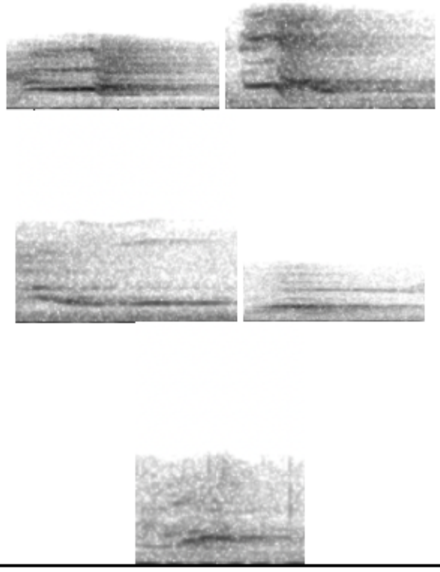
D<br><br>Chips	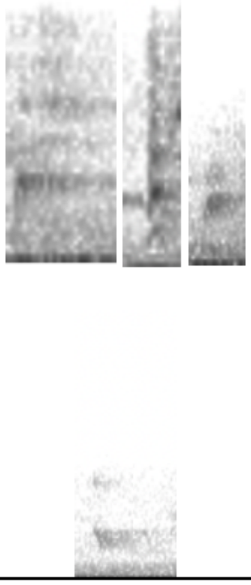	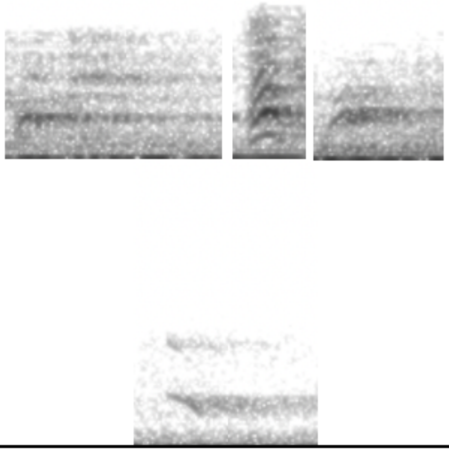

E<br><br>Flat-fronted harmonic stacks (sometimes with low frequency whistle)	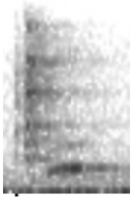<br>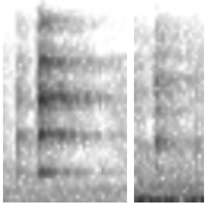<br>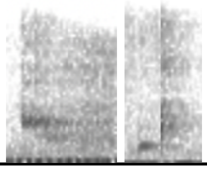	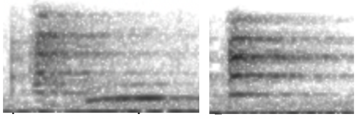<br>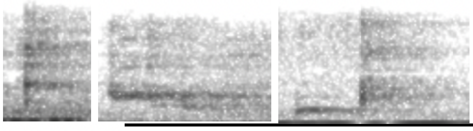
F<br><br>Flat harmonic stack	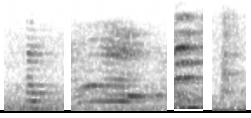	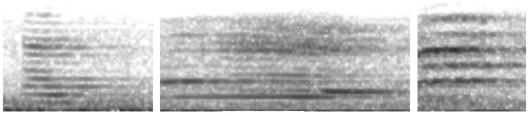<br>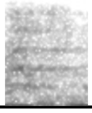

G<br>Upward frequency modulation to flat harmonic stack to prominent low frequency trace	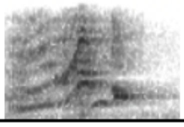	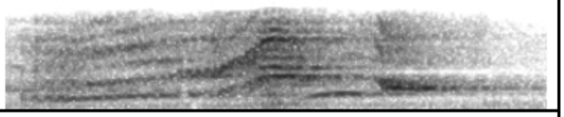
H<br><br>Upward plateaued frequency modulation	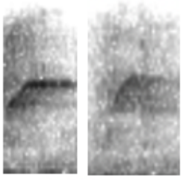<br>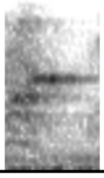	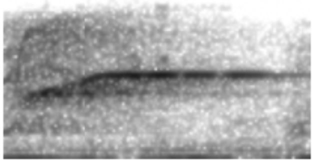<br>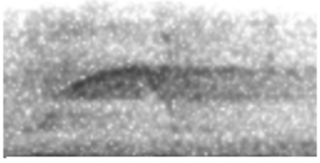<br>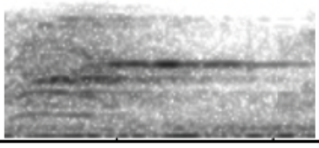

I<br><br>Upward frequency modulation to flat harmonic stack	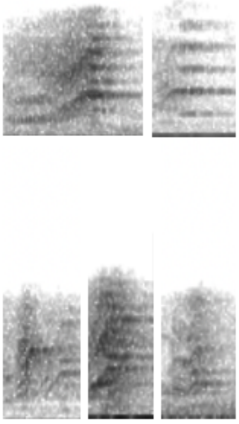	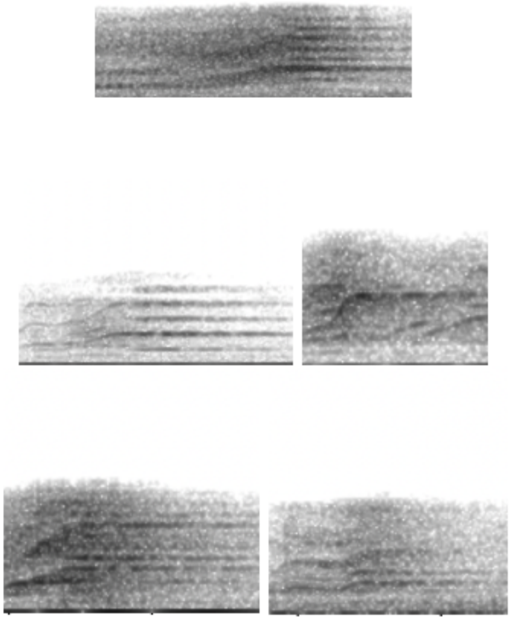
J<br><br>Upward frequency modulation with multiple traces	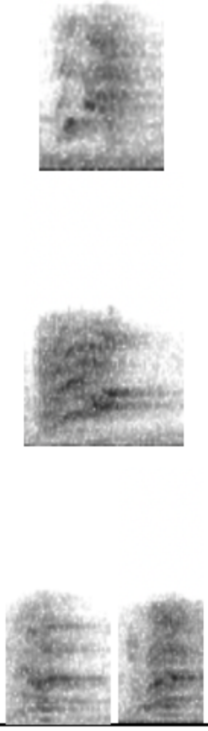	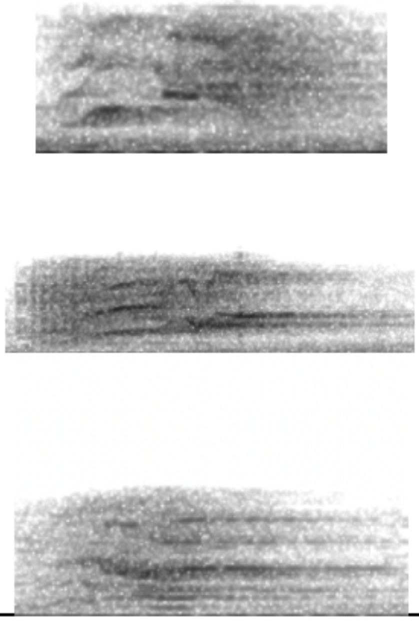

		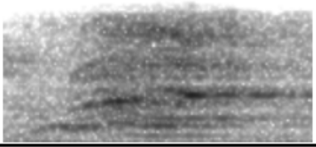
K<br><br>Curved downward frequency modulation with low subharmonic	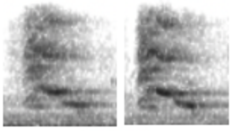<br>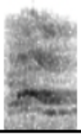	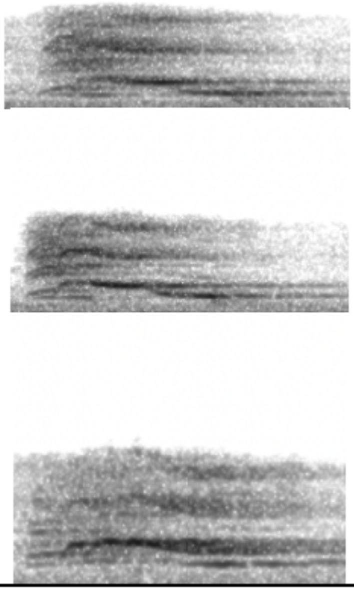

L<br><br>Curved downward frequency modulations	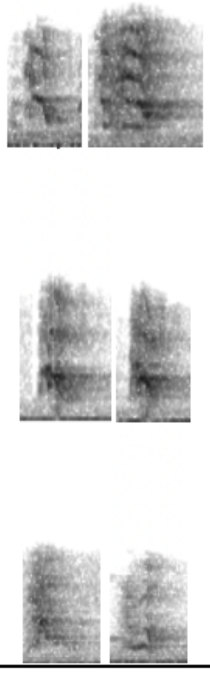	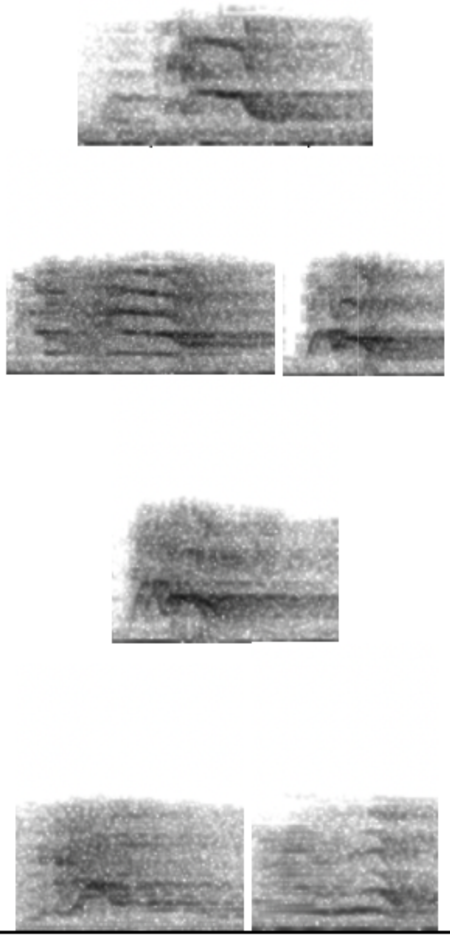
M<br><br>Downward plateaued modulation with narrow harmonics	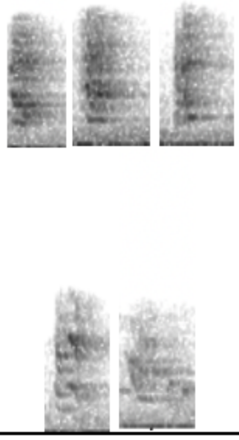	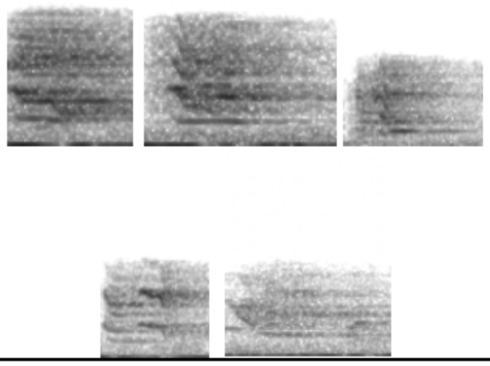

N<br><br>Flat frequency harmonic stack to chaotic harmonic stack	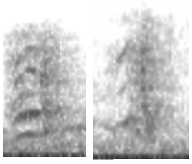<br>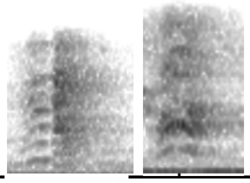	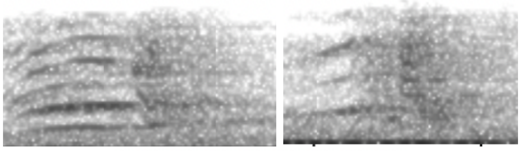<br>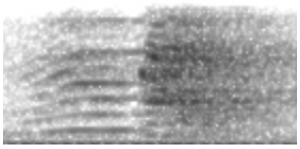<br>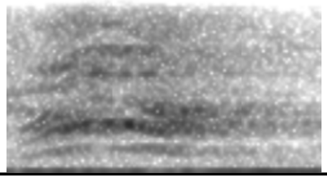
O<br><br>Noisy harmonic stack	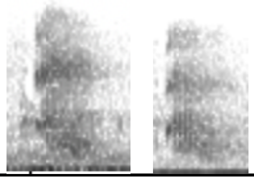	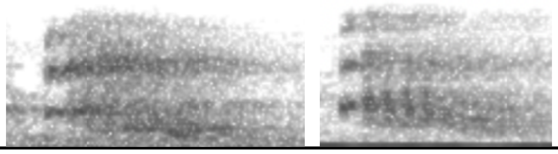

P<br><br>Downward frequency modulation with noisy harmonics	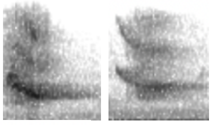<br>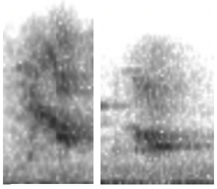<br>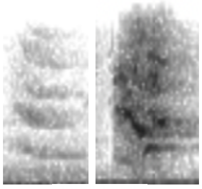<br>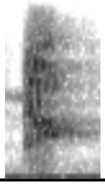	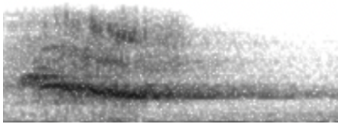<br>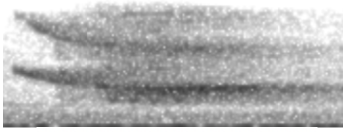<br>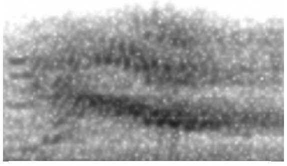<br>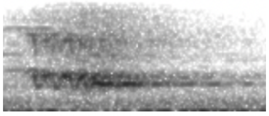<br>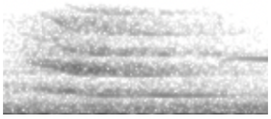<br>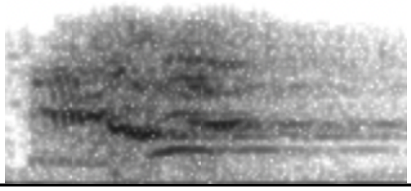

Q<br><br>Harmonic stack (narrow spacing) to harmonic stack (wide spacing)	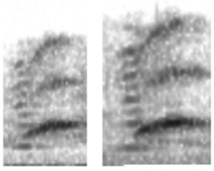<br>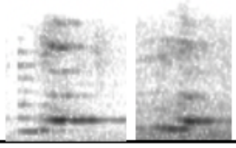	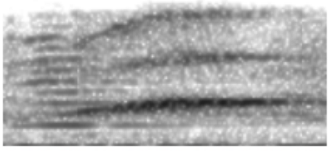<br>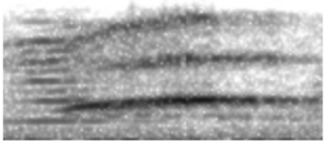<br>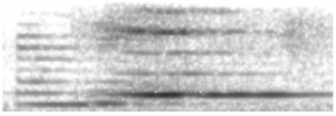<br>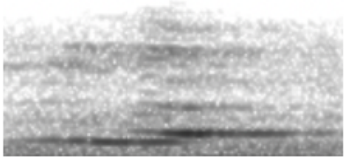

R<br><br>Notched harmonic stacks	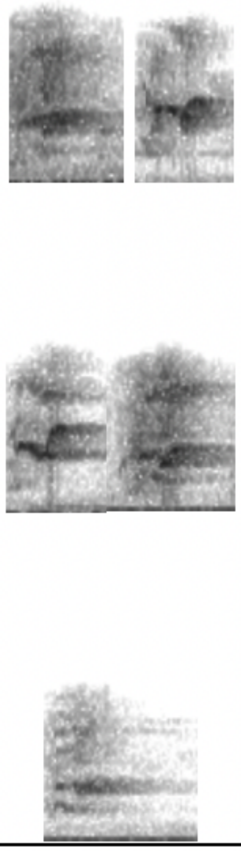	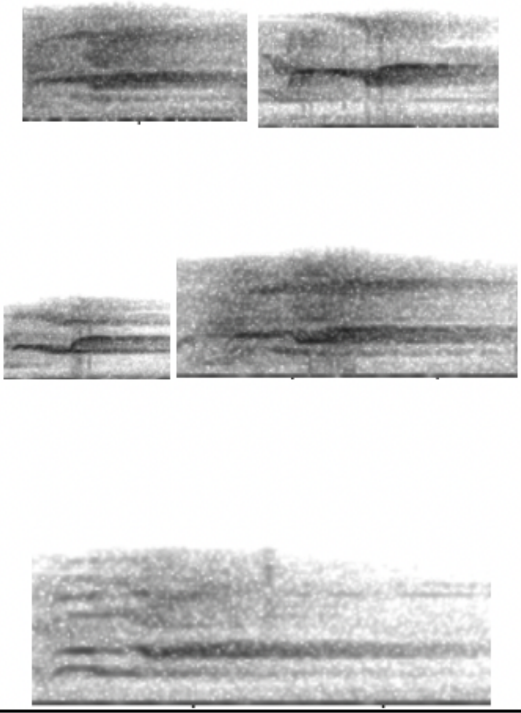
S<br><br>Harmonic Inverted ‘U’ modulation	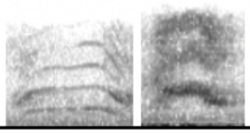	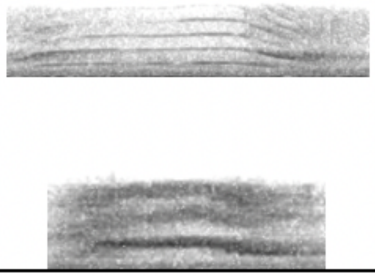
T<br><br>Noisy harmonic stack	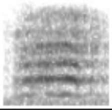	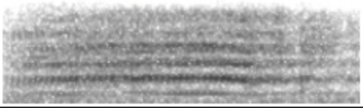

U<br><br>Rattles and trills	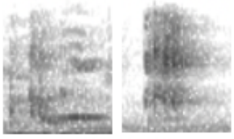<br>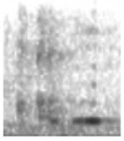<br>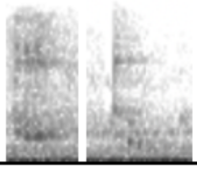	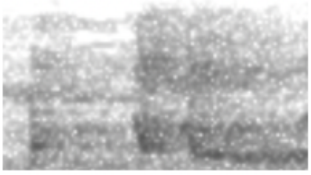<br>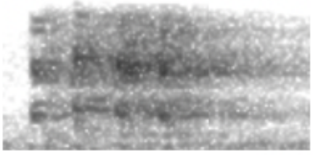<br>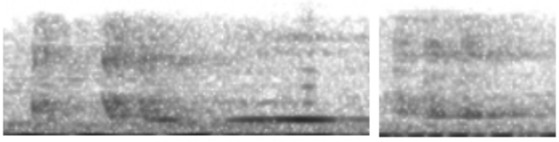<br>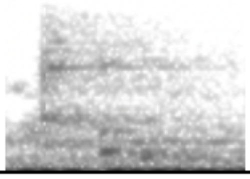

V<br>Curved downward frequency modulation with middle flat	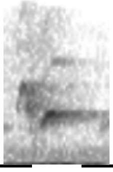	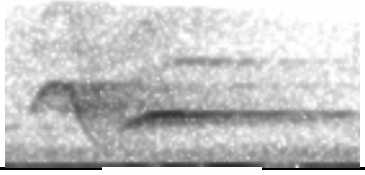
W<br>Flat middle modulations	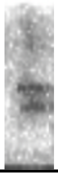	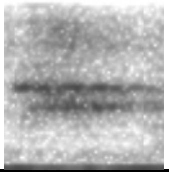
